# Transfer of cognitive control adjustments within and between speakers

**DOI:** 10.1177/17470218241249471

**Published:** 2024-05-17

**Authors:** Paul Kelber, Ian Grant Mackenzie, Victor Mittelstädt

**Affiliations:** University of Tübingen, Tübingen, Germany

**Keywords:** Congruency sequence effect, cognitive control, transfer, abstract context, binding and retrieval, confound-minimised design

## Abstract

Congruency effects in conflict tasks are typically larger after congruent compared to incongruent trials. This congruency sequence effect (CSE) indicates that top-down adjustments of cognitive control transfer between processing episodes, at least when controlling for bottom-up memory processes by alternating between stimulus-response (S-R) sets in confound-minimised designs. According to the control-retrieval account, cognitive control is bound to task-irrelevant context features (e.g., stimulus position or modality) and retrieved upon subsequent context feature repetitions. A confound-minimised CSE should therefore be larger when context features repeat rather than change between two trials. This study tested this prediction for a more abstract contextual stimulus feature, speaker gender. In two preregistered auditory prime-probe task experiments, participants classified colour words spoken by a female or male voice. Across both experiments, we found confound-minimised CSEs that were not reliably affected by whether the speaker gender repeated or changed. This indicates that speaker transitions have virtually no influence on the transfer of control adjustments in the absence of S-R repetitions. By contrast, when allowing for bottom-up memory processes by repeating the S-R set, CSEs were consistently larger when the speaker gender repeated compared to changed. This suggests that speaker transitions can in principle influence transfer between processing episodes. The discrepancy also held true when considering learning and test episodes separated by an intervening episode. Thus, the present findings call for a refinement of the control-retrieval account to accommodate the role of more abstract contextual stimulus features for the maintenance of memory traces in auditory conflict processing.

## Introduction

Adaptive goal-directed behaviour requires continual adjustments in the allocation of one’s limited cognitive resources to incoming information. To exert such cognitive control, one has to flexibly focus on goal-relevant information and ignore goal-irrelevant information based on the current situational demands. It is thus central for our understanding of cognitive control to uncover how control settings adapted in one situation are transferred to other situations. The discussion of how such transfer takes place is increasingly concerned with the role of abstract representations, that is, higher-level mental states (e.g., a task goal or an inferred category) that are by and large independent of specific (lower-level) stimulus and response modalities. Such abstract representations have recently been shown to play an important role in orchestrating transfer (e.g., Horner & Henson, 2009, 2011; Longman et al., 2018, 2019, 2020, 2023; Pfeuffer et al., 2018). This study aims to extend this research by examining how the transfer of cognitive control adjustments in the presence of multiple, potentially conflicting sources of information (in a conflict task) depends on whether abstract representations of the context match or mismatch between the learning episode and the test episode. Specifically, we investigated to what extent control settings that are generated during the experience of auditory conflict conveyed by one speaker (e.g., a male voice) transfer to subsequent processing of conflict conveyed by another speaker (e.g., a female voice).

The extent to which people exert cognitive control is often studied in conflict tasks (e.g., Eriksen & Eriksen, 1974; Simon, 1969; Stroop, 1935), in which participants have to classify a target irrespective of a potentially interfering or facilitating distractor. An exemplary conflict task is one version of the prime-probe task (e.g., Dehaene et al., 1998; Greenwald et al., 1996; Kunde et al., 2003; Vorberg et al., 2003), in which a distractor (or prime) stimulus is presented shortly before a target (or probe) stimulus. In this prime-probe task, participants have to respond to one dimension of the target (e.g., colour). Crucially, this target feature is manipulated orthogonally to an associated distractor feature (e.g., red or blue colour, respectively) across trials, thus yielding congruent trials (e.g., blue distractor followed by blue target) and incongruent trials (e.g., red distractor followed by blue target). Performance—as measured by response time (RT) and/or error rate (ER)—is usually worse in incongruent trials (when distractor and target suggest different responses) than in congruent trials (when distractor and target suggest the same response). This *congruency effect* (CE = RT_incongruent_ − RT_congruent_ or ER_incongruent_ − ER_congruent_) is often interpreted as a marker of cognitive control, defined as the extent to which processing is focused on the target relative to the distractor (e.g., Botvinick et al., 2001).

The CE is typically larger in trials following a congruent trial than in trials following an incongruent trial (Gratton et al., 1992; Kerns et al., 2004; Kunde & Wühr, 2006; Stürmer et al., 2002). This *congruency sequence effect* (CSE = CE_previous congruent_ − CE_previous incongruent_) can be seen as a marker of control adjustments, that is, adaptations of target-to-distractor processing. Accordingly, the CSE reflects stronger target processing and/or weaker distractor processing after incongruent trials than after congruent trials.

However, explanations of the CSE in terms of control adjustments (e.g., Botvinick et al., 2001; Gratton et al., 1992; Ridderinkhof, 2002) have been challenged by the lucid observation that congruency sequence is confounded with feature transition in ordinary two-alternative forced-choice (2AFC) designs (Hommel et al., 2004; Mayr et al., 2003). Specifically, task-related stimulus or response (S-R) features completely repeat or completely alternate when congruency repeats between two trials (congruent → congruent, or alternatively, incongruent → incongruent), whereas those features partially repeat when congruency changes (congruent → incongruent, or alternatively, incongruent → congruent). Based on this confound, Hommel et al. (2004) developed a *feature-integration account* of the CSE derived from the theory of event coding (Hommel et al., 2001), according to which the representations of perceived and to-be-produced events share a common code. It is further assumed that co-occurring features are bound together in a transient representational structure and retrieved upon subsequent activation of one of its constituent features. Therefore, partial feature repetitions hamper performance compared to full feature repetitions or alternations, since invalid associations must be dissolved. As a result, the CSE in such confounded designs may also reflect partial-repetition costs instead of only control adjustments.

Moreover, by increasing the number of stimuli and responses to rule out S-R repetitions, contingency biases can be introduced, where a specific distractor is more often associated with the congruent target than with any specific incongruent target. In this case, congruency transition is confounded with contingency transition. According to the *contingency-learning account* (Schmidt, 2013; Schmidt & De Houwer, 2011; see also Mordkoff, 2012), the contingency transition can produce a CSE, since the “contingency effect”—impaired performance for low-contingency (incongruent) trials compared to high-contingency (congruent) trials—has been shown to be larger after high-contingency (congruent) trials than after low-contingency (incongruent) trials.

In summary, the theoretical accounts of the CSE can essentially be divided as to whether they assume that this effect arises due to lower-level memory-based learning processes (e.g., associative learning of S-R links) and/or due to higher-level cognitive control processes. To isolate control-based effects from memory-based effects due to feature integration or contingency learning, confound-minimised designs can be used, which prevent S-R repetitions between consecutive trials, while keeping the proportion of congruent and incongruent trials in balance (Braem et al., 2019; Jiménez & Méndez, 2013; Kim & Cho, 2014; Schmidt & Weissman, 2014). One such option is to alternate between two 2AFC tasks that are characterised by distinct S-R sets. Confound-minimised prime-probe tasks have consistently yielded a CSE, which can be more easily attributed to control adjustments compared to the CSE in confounded designs (e.g., Gyurkovics et al., 2020; Schmidt & Weissman, 2014; Weissman et al., 2014, 2015).

Critically, an extension of the feature-integration account, the *control-retrieval account*, can also explain this confound-minimised CSE (e.g., Dignath et al., 2019; Egner, 2014; Jiang et al., 2015; Schumacher & Hazeltine, 2016; Spapé & Hommel, 2008; Weissman et al., 2016; Whitehead et al., 2020, 2022). Accordingly, binding and retrieval processes are not limited to S-R features but also pertain to the context and cognitive control. Thus, even in the absence of S-R repetitions, a CSE can arise as follows: In the wake of trial n−1 , cognitive control is adjusted based on the detected conflict and this adjusted level of cognitive control is bound to the encountered context (i.e., to features that are task-irrelevant but nevertheless processed such as the spatial position of the distractor and the target in the prime-probe task). In trial n , a context repetition (such as the same stimulus position) causes the retrieval of the bound cognitive control. The stronger these binding and retrieval processes (e.g., the more context features repeat), the stronger the transfer of control adjustments from trial n−1 to trial n (i.e., the larger the CSE). Therefore, the control-retrieval account predicts that the confound-minimised CSE is larger when a context feature repeats (vs. changes) between consecutive trials.

This prediction has been confirmed in several confound-minimised prime-probe-like tasks. Specifically, a larger CSE was observed for repetitions (vs. changes) of lower-level features of distractor and target, such as stimulus modality (Grant et al., 2020; Grant & Weissman, 2023; Kelber et al., 2023), format (Dignath et al., 2019; Grant et al., 2022; Schiltenwolf et al., 2024), and position (Dignath & Kiesel, 2021). Taken together, converging evidence seems to support the control-retrieval account, according to which the transfer of control adjustments between two tasks with distinct S-R sets is mediated by context similarity (see also Braem et al., 2014).

However, it is still largely unclear which types of contextual stimulus features guide the transfer of control adjustments. One way to classify contextual stimulus features is in terms of their “abstractness.” According to our working definition, more concrete stimulus features (e.g., position) consist in lower-level characteristics of the input signal, whereas more abstract stimulus features (e.g., gender) consist in higher-level characteristics that must be inferred based on the input signal (Kelber et al., 2023). Most previous studies (see above) manipulated the context via a concrete (lower-level) stimulus feature such as modality (e.g., written or spoken direction words), format (numbers represented as words or digits), or position (bottom or top). Thus, it seems that the transfer of control adjustments is mediated by the transition of concrete features of proximal stimulation. This could be due to adaptations in the processing strength of lower-level perceptual channels, for instance.

To test whether the prediction of the control-retrieval account also holds for the transition of more abstract (higher-level) stimulus features, we recently manipulated stimulus intensity (weak vs. strong) while alternating between visual and auditory stimuli (Kelber et al., 2023). A larger CSE was observed when the cross-modal intensity of the distractor and target stimuli repeated (e.g., bright → loud) compared to changed (e.g., bright → soft). This complements psychophysical studies showing that perceived brightness of a light is enhanced by a concurrent tone (Stein et al., 1996), and that perceived loudness of a tone is enhanced by a concurrent light (Odgaard et al., 2004). Furthermore, Johansson et al. (2024) argued that the audio-visual intensity correspondence reflects an adaptation to statistical regularities observed in the environment. Taking this into account, the observation by Kelber et al. (2023) seems to suggest that the transfer of control adjustments is also mediated by the transition of more abstract stimulus features originating from statistical correspondences during early multisensory integration.

In general, however, more abstract stimulus features have received little attention as context features so far. Only Spapé and Hommel (2008) assessed whether the CSE is modulated by the transition of a contextual stimulus feature that seems to carry more semantically relevant information about the distal stimulus. They tasked 14 participants to classify a pure (sine) tone as low-pitched (550 Hz) or high-pitched (1,050 Hz) via vocal response (“low” or “high” in Dutch). Concurrent to the presentation of the target tone, the Dutch word for “low” or “high” was spoken by a female, male, or the participant’s voice (pre-recorded before the experiment). In this auditory Stroop task, the CSE depended critically upon whether the speaker of the distractor tone repeated or changed between consecutive trials, with the CSE being present for speaker repeats (76 ms) but smaller and absent for speaker changes (4 ms). This seems to suggest that people adapt the relative activation generated by the spoken distractor word (compared to the target tone) more strongly to the just experienced conflict between the distractor word and the target tone when the distractor words are spoken by the same person (vs. by different persons).

Thus, the transfer of control adjustments might also be mediated by the transition of more abstract stimulus features that provide rich information for later semantic processing (here: speaker gender). However, an interpretation of this result in terms of control adjustments is open to attack because S-R repetitions were not precluded (i.e., the design was confounded). This makes it impossible to untangle whether the speaker transition affected the transfer of control adjustments and/or S-R-based binding and retrieval processes. Specifically, the larger CSE for speaker repeats (vs. changes) might have been due to an enhanced transfer of control adjustments and/or to an enhanced influence of S-R feature transitions. The latter seems plausible, considering that the transition of the task-irrelevant speaker gender has been shown to interact with the response transition (Bogon et al., 2017; Herwig & Waszak, 2012). Therefore, as of now, it is unclear whether more abstract and semantically relevant contextual stimulus features guide the transfer of control adjustments between S-R sets.

In this study, two preregistered experiments assessed whether the CSE is modulated by the transition of the speaker gender (female, male) in a confound-minimised auditory prime-probe task. In this way, we could assess whether the transfer of control adjustments depends on whether an abstract context representation (speaker gender) repeats or changes from the learning episode to the test episode. A prime-probe task was used for comparability with previous confound-minimised studies and because this task has been shown to yield reliable CSEs and CSE modulations by context transition in the absence of S-R repetitions (see above). Auditory stimuli were used for comparability with the study by Spapé and Hommel (2008), because the speaker gender seemed more salient to us in audition than in vision, and because an auditory stimulus feature has not been considered as context in previous assessments of the confound-minimised CSE. In Experiment (Exp.) 1, we used a confound-minimised design in which two 2AFC prime-probe tasks with distinct S-R sets were alternating every trial (i.e., A–B–A–B). By using this confound-minimised design, we could isolate the transfer of control adjustments from memory-based confounds due to feature integration and contingency learning. Thus, Exp. 1 provided a first direct test of whether abstract context representations (speaker gender) can guide cognitive control adjustments in resolving conflict. In Exp. 2, two 2AFC prime-probe tasks were alternating every *other* trial (i.e., A–A–B–B), enabling us to directly contrast the CSE modulation by context transition for confound-minimised and confounded transitions from trial n−1 to trial n . In doing so, Exp. 2 allowed us to compare transfer within and between S-R sets under otherwise identical conditions.

In addition to examining the CSE defined by the congruency in adjacent trials n−1 and n (i.e., the CSE_*n*–1_), we also looked at the CSE defined by the congruency in the trials n−2 and n (i.e., the CSE_*n*–2_). A CSE_*n*–2_, which has already been observed in some studies (e.g., Jiménez & Méndez, 2013; Kim & Cho, 2014; Lee & Cho, 2013; Mayr et al., 2003), would point to a “far” transfer of processing characteristics between non-adjacent episodes. Furthermore, a modulation of the CSE_*n*–2_ by the transition of a context feature (here: speaker gender), which has to our knowledge not been reported yet, would suggest that information about the context feature and the associated features can be retained for longer durations and affect subsequent performance despite an intervening trial. As a result of the different S-R set transitions in the two experiments, the transition from trial n−2 to trial n was confounded in Exp. 1 but confound-minimised in Exp. 2. Thus, in Exp. 1, the confounded CSE_*n–*2_ allowed us to explore the role of abstract context representations for transfer from the learning episode to a later test episode within the same S-R set. In Exp. 2, however, the confound-minimised CSE_*n*–2_ allowed us to examine the transfer of control adjustments across an intervening trial and across distinct S-R sets.

## Experiment 1

Exp. 1 assessed the speaker dependence of the CSE in a confound-minimised auditory prime-probe task where participants had to classify a spoken colour word (target) irrespective of a preceding spoken colour word (distractor). Distractors, targets, and correct responses were not allowed to repeat between consecutive trials, whereas the speaker gender could either repeat (e.g., female → female) or change (e.g., female → male). This enabled us to study whether the transfer of control adjustments depends on a more abstract contextual stimulus feature. Specifically, the results of Exp. 1 should differentiate between three potential accounts of the transfer of control adjustments between S-R sets: (1) complete, (2) partial, and (3) no dependence on the transition of the speaker gender. First, if a transfer of control adjustments between S-R sets takes place within but not between abstract contexts, the confound-minimised CSE should be larger for speaker gender repeats than for speaker gender changes and absent in the latter case. Second, if the transfer of control adjustments between S-R sets is stronger within (vs. between) abstract contexts but still exists between abstract contexts, the confound-minimised CSE should again be larger for speaker gender repeats (vs. changes) and still be present in the latter case. Third, if the transfer of control adjustments between S-R sets is not sensitive to the transition of abstract contexts, the confound-minimised CSE should not differ between speaker gender repeats and changes.

In contrast to the confound-minimised trial transition n−1→n , the trial transition n−2→n comprised S-R repetitions. By repeating the analyses for the trial transition n−2→n , we could thus explore potential differences between (1) the transfer of control adjustments across different S-R sets and (2) the transfer of adjustments of cognitive control or S-R-related binding and retrieval processes within the same S-R set. Here, too, three scenarios are conceivable: Transfer within S-R sets (1) may only take place within but not between abstract contexts, (2) may be larger within than between abstract contexts but still exist in the latter case, or (3) may not depend on the abstract context.

### Method

#### Participants

A sample of 50 participants from the student pool at the University of Tübingen was tested online. According to an *a priori* power analysis, this sample size ensures a statistical power of at least 1−β=.80 (using a significance level of α=.05 ) to detect our smallest effect size of interest for the CSE modulation by speaker transition in mean RTs ( $\eta_{p}^{2}=.15$ ). This is the smallest effect size detected across a wide range of previous studies assessing CSE modulations by other context transitions in prime-probe tasks (Dignath et al., 2019, Exp. 1–2; Grant et al., 2020, Exp. 1; Grant et al., 2022, Exp. 1; Grant & Weissman, 2023, Exp. 1–3; Hazeltine et al., 2011, Exp. 1; Kelber et al., 2023, Exp. 1–2). Accordingly, we deem CSE modulations by speaker transition as negligible if and only if they fail to reach the size of the smallest comparable effect detected in previous studies.

All participants in this and the following experiment provided informed consent prior to testing and received course credit or financial reimbursement (£5) for attending one session that lasted about 40 min. Both experiments were in accordance with the ethical standards of the institutional and national research committee as well as with the 1964 Helsinki Declaration and its later amendments or comparable ethical standards. In Exp. 1, the data from one participant were excluded from all analyses due to an ER above 25% (27.3%; all other ERs < 13.9%), which did not affect any statistical inference. The final sample thus comprised 49 participants (46 female, 40 right-handed, mean age: 20.9, age range: 18–30 years).

#### Apparatus, stimuli, and procedure

Stimulus presentation and response recording were controlled using the *Java-Script* library *jsPsych* (de Leeuw, 2015). Distractors and targets were German words for red (“rot”), green (“grün”), blue (“blau”), and yellow (“gelb”) spoken by a digital female and male voice and edited to last for 300 ms. The left-side keys “Q” and “W” on the participants’ keyboards served as response devices in one 2AFC task, and the right-side keys “O” and “P” in the other. RT was measured from target onset to the first key press registration. To avoid feature-integration and contingency-learning confounds, two 2AFC tasks with distinct S-R sets (e.g., {red–Q, green–W}, {blue–O, yellow–P}) alternated between consecutive trials. These S-R sets were randomly generated for each participant. In total, there were eight different trial types (two S-R sets × two distractors × two targets). The trials within each block were randomised with the constraint that the two S-R sets had to alternate.

**Figure 1.**
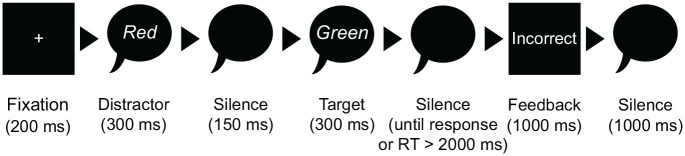
Example trial course. *Note.* In the example trial shown above, distractor (*red*) and target (*green*) were incongruent, and the response was incorrect. Speech bubbles represent auditory stimuli, and squares represent visual stimuli. If an empty speech bubble is displayed, there was no sound. Likewise, if no square is displayed, the screen was blank. A feedback screen was only shown when the response was not correct or not given in the response window.

Each participant completed 12 blocks, the first of which was practice. One block comprised 64 trials (eight repetitions of eight trial types). As can be seen in Figure 1, each trial started with the presentation of a white fixation cross for 200 ms in the centre of the screen against a black background. It was followed by the spoken distractor word (300 ms), silence (150 ms), and the spoken target word (300 ms). The screen remained blank from distractor onset to the participant’s response or till RT exceeded 2,000 ms. Visual feedback was displayed along with the S-R mapping for 1,000 ms if the response was incorrect (“Falsch!”) or not given within the response window of 2,000 ms (too slow; “Zu langsam!”). The S-R mapping was shown by colour-coding the names of the four response keys (e.g., “Q key” written in red letters, “W key” written in green letters, etc.). The trial ended after another blank screen (1,000 ms).

### Results and discussion

**Figure 2.**
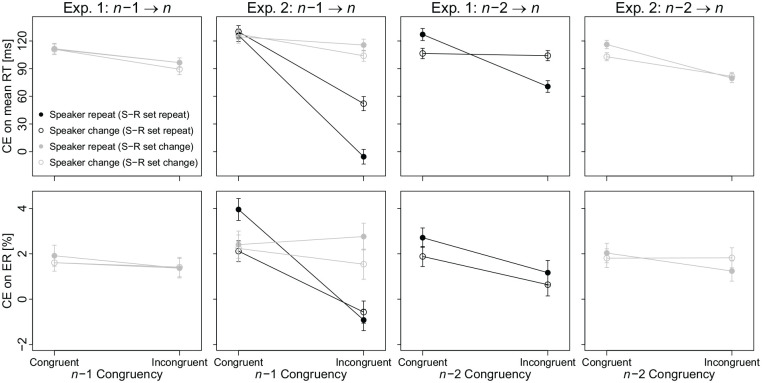
CE on mean RT (first row) and ER (second row) in Exp. 1 (first and third columns) and Exp. 2 (second and fourth columns) as a function of previous congruency (congruent vs. incongruent), speaker transition (repeat vs. change), and S-R set transition (repeat vs. change). *Note.* Error bars represent ± 1 within-subjects standard error of the mean according to the method by Cousineau (2005) with the correction from Morey (2008).

We excluded trials with an RT below 150 ms (0.1%), trials in which no response was given within 2,000 ms (0.9%), and trials that followed an incorrect response (4.1%). Furthermore, trials with an incorrect response (4.1%) were excluded from all RT analyses. Based on mean RTs and ERs for the remaining trials, a CE was calculated. The participants’ CEs were subjected to separate two-way repeated-measures analyses of variance (ANOVAs) with the factors n−1 *congruency* (congruent vs. incongruent), and *speaker transition* (repeat vs. change).^1^ We also directly assessed whether CSEs were present separately for speaker repeats and changes using one-sample *t*-tests, and whether CSEs differed for speaker repeats and changes using a paired *t*-test. The main results can be gleaned from Figure 2.

For CEs in mean RTs, there was a significant main effect of n−1 *congruency*, F(1,48)=11.00 , p=.002 , $\eta_{p}^{2}=.19$ , reflecting a CSE (111–93 = 18 ms). This effect indicates a transfer of control adjustments between different S-R sets. No other effect was significant (both *p*s > .469). In particular, the CSE was significant for speaker changes (22 ms), t(48)=2.57 , p=.013 , $d_{z}=0.37$ , not quite for speaker repeats (15 ms), t(48)=1.94 , p=.058 , $d_{z}=0.28$ , and did not differ significantly between speaker repeats and changes, t(48)=−0.55 , p=.582 , $d_{z}=-0.08$ . Contrary to the prediction of the control-retrieval account, this does not indicate that the transfer of control adjustments between S-R sets is larger for speaker repeats (vs. changes). For CEs in ERs, there was no significant effect (all *p*s > .426).

To explore whether the CSE depends on the speaker transition in a confounded setting, we repeated the same analyses for the ${\mathrm{CSE}}_{n-2}$ . In these transitions, stimuli and responses can repeat, which is why the ${\mathrm{CSE}}_{n-2}$ likely involves lower-level memory-based learning processes and hence cannot be seen as pure marker of higher-level cognitive control adjustments. For these analyses, we additionally removed the second trial from each block and trials for which an error occurred in trial n−2 (4.4%). Mean RTs and ERs were then subjected to separate two-way repeated-measures ANOVAs with the factors n−2 *congruency* and *speaker transition*. For CEs in mean RTs, a significant main effect of n−2 *congruency*, F(1,48)=20.85 , p<.001 , $\eta_{p}^{2}=.30$ , reflects a ${\mathrm{CSE}}_{n-2}$ (117 – 87 = 30 ms). This indicates a transfer of control adjustments and/or S-R-based binding and retrieval processes within S-R sets from trial n−2 to trial n . The main effect of *speaker transition* was not significant, F(1,48)=1.29 , p=.261 , $\eta_{p}^{2}=.03$ . Interestingly, there was a significant interaction between n−2 *congruency* and *speaker transition*, F(1,48)=20.30 , p<.001 , $\eta_{p}^{2}=.30$ . In complete contrast to the n−1 analysis, the ${\mathrm{CSE}}_{n-2}$ was not significant for speaker changes (2 ms), t(48)=0.30 , p=.762 , $d_{z}=0.04$ , but for speaker repeats (56 ms), t(48)=5.85 , p<.001 , $d_{z}=0.84$ , and significantly larger for speaker repeats than for speaker changes, t(48)=4.51 , p<.001 , $d_{z}=0.64$ . This indicates that the transfer from trial n−2 to trial n was stronger for speaker repeats (vs. changes). For CEs in ERs, there was only a significant main effect of n−2 *congruency* ${\mathrm{(CSE}}_{n-2}=2.3-0.9=1.4\%)$ , F(1,48)=8.26 , p=.006 , $\eta_{p}^{2}=.15$ (both other *p*s > .193).^2^

In summary, Exp. 1 provided evidence for the transfer of cognitive control adjustments (as measured by the confound-minimised CSE), but it did not suggest that this transfer was further modulated by the transition of the speaker gender. However, an additional finding suggested that this transition can influence the transfer across processing episodes. Specifically, the transition of speaker gender from trial n−2 to trial n affected the confounded CSE (larger CSE for speaker repeats than for speaker changes), which can reflect control- and/or memory-based learning processes.

## Experiment 2

In Exp. 1, the confound-minimised ${\mathrm{CSE}}_{n-1}$ was not reliably affected by whether the speaker gender repeated or changed. This result is inconsistent with the prediction of the control-retrieval account. Instead, it suggests that control adjustments transfer between S-R sets irrespective of whether the learning episode and the test episode share the same abstract context. Interestingly, the transition of speaker gender did influence transfer from trial n−2 to trial n , with the confounded ${\mathrm{CSE}}_{n-2}$ being larger for speaker gender repeats (vs. changes).

Two differences between the ${\mathrm{CSE}}_{n-1}$ and ${\mathrm{CSE}}_{n-2}$ lend themselves as candidate explanations for these divergent outcomes. One difference is that the trial transition n−2→n included S-R repetitions, while the trial transition n−1→n transition did not. Accordingly, the speaker transition might only affect S-R-set-specific transfer of control adjustments (e.g., Hazeltine et al., 2011) or even only S-R-based binding and retrieval processes (e.g., Hommel et al., 2004; Mayr et al., 2003). This S-R set specificity account is consistent with the finding by Berger et al. (2020) with regard to the proportion congruency effect (PCE; Logan & Zbrodoff, 1979), which consists in a smaller CE for higher proportions of incongruent compared to congruent trials. In an auditory Simon task, Berger et al. (2020) found a smaller CE for the speaker gender associated with mostly incongruent trials than for the speaker gender associated with mostly congruent trials. In line with an S-R set specificity account, this PCE occurred for frequency-biased but not for frequency-unbiased items. However, dissociations between the CSE and the PCE point to different underlying processes (e.g., Funes et al., 2010; Wühr et al., 2015) and thus limit comparability. Another potentially relevant difference between the ${\mathrm{CSE}}_{n-1}$ and ${\mathrm{CSE}}_{n-2}$ is that the trial transition n−2→n provided participants with more time to process the speaker information than the trial transition n−1→n . Possibly, the speaker information needed this additional time to ascend towards long-term memory to some degree in order to bias the amount of control in trial n (temporal account).

The aim of Exp. 2 was to disentangle the S-R-set specificity account and the temporal account of the divergent results of the n−1 and n−2 analyses in Exp. 1. To this end, we changed the S-R set transition from A–B–A–B to A–A–B–B. This allowed us to directly compare how the transition of speaker gender affects the confounded and the confound-minimised ${\mathrm{CSE}}_{n-1}$ . Specifically, if the speaker transition only affects S-R-set-specific transfer of control adjustments and/or S-R-based binding and retrieval, speaker transition should modulate the confounded ${\mathrm{CSE}}_{n-1}$ , but neither the confound-minimised ${\mathrm{CSE}}_{n-1}$ nor the confound-minimised ${\mathrm{CSE}}_{n-2}$ . Furthermore, as the ${\mathrm{CSE}}_{n-2}$ now always involved switches between S-R sets (i.e., the ${\mathrm{CSE}}_{n-2}$ was confound-minimised), we could also examine whether the speaker transition modulates the transfer of control adjustments between learning episode and test episode when they are separated by an intervening episode. Specifically, if the speaker transition only affects the transfer of control adjustments for longer durations between learning and transfer episode, the speaker transition should modulate the confound-minimised ${\mathrm{CSE}}_{n-2}$ , but neither the confounded nor the confound-minimised ${\mathrm{CSE}}_{n-1}$ .

### Method

#### Participants

A sample of 80 participants consisting of University of Tübingen students and Prolific workers was tested online. We decided on this increment compared to the 50 participants in Exp. 1 considering the inclusion of an additional independent variable (new design of Exp. 2: n−1 *congruency* × *speaker transition* × *S-R set transition*) and the resources available to us. The data from one participant were excluded from all analyses due to an ER above 25% (50.5%; all other ERs < 23.0%). The final sample thus comprised 79 participants (44 female, 72 right-handed, mean age: 25.0, age range: 18–40 years).

#### Apparatus, stimuli, and procedure

The only difference to Exp. 1 was that S-R sets did not alternate every trial (A–B–A–B), but every other trial (A–A–B–B).

### Results and discussion

4.7% of trials followed an incorrect response, 0.1% of trials included an anticipatory response, 0.5% included no response, and 4.7% included an incorrect response. For the analysis of the ${\mathrm{CSE}}_{n-1}$ , CEs in mean RTs and ERs (see Figure 2) were subjected to separate three-way repeated-measures ANOVAs with the factors n−1 *congruency* (congruent vs. incongruent), *speaker transition* (repeat vs. change), and *S-R set transition* (repeat vs. change).

For CEs in mean RTs, all effects in the three-way ANOVA were significant. The main effect of n−1 *congruency*, F(1,78)=117.23 , p<.001 , $\eta_{p}^{2}=.60$ , reflects a CSE (127 – 67 = 60 ms), thus indicating transfer from trial n−1 to trial n . Furthermore, main effects of *speaker transition*, F(1,78)=9.23 , p=.003 , $\eta_{p}^{2}=.11$ , and *S-R set transition*, F(1,78)=56.75 , p<.001 , $\eta_{p}^{2}=.42$ , reflect a larger CE for speaker changes (103 ms) than for speaker repeats (90 ms), and a larger CE for S-R set changes (118 ms) than for S-R set repeats (76 ms), respectively. Two-way interactions of n−1 *congruency* and *speaker transition*, F(1,78)=6.73 , p=.011 , $\eta_{p}^{2}=.08$ , n−1 *congruency* and *S-R set transition*, F(1,78)=78.99 , p<.001 , $\eta_{p}^{2}=.50$ , as well as *speaker transition* and *S-R set transition*, F(1,78)=16.27 , p<.001 , $\eta_{p}^{2}=.17$ , reflect a larger CSE for speaker repeats (70 ms) than for speaker changes (51 ms), a larger CSE for S-R set repeats (105 ms) than for S-R set changes (16 ms), as well as a larger CE modulation by speaker transition for S-R set repeats (speaker repeat: 60 ms, change: 91 ms) than for S-R set changes (speaker repeat: 120 ms, change: 116 ms).

Most importantly, the three-way interaction was significant, F(1,78)=12.44 , p=.001 , $\eta_{p}^{2}=.14$ , reflecting a larger CSE modulation by speaker transition for S-R set repeats (vs. changes). This indicates that the transfer from trial n−1 to trial *n* was more pronounced within (vs. between) S-R sets. Specifically, for S-R set repeats, the CSE was significantly larger for speaker repeats (132 ms; t(78)=12.23 , p<.001 , $d_{z}=1.38$ ) than for speaker changes (78 ms; t(78)=7.76 , p<.001 , $d_{z}=0.87$ ), t(78)=4.35 , p<.001 , $d_{z}=0.49$ . By contrast, for S-R set changes, the CSE did not differ significantly between speaker repeats (9 ms; t(78)=0.97 , p=.337 , $d_{z}=0.11$ ) and speaker changes (23 ms; t(78)=2.74 , p=.008 , $d_{z}=0.31$ ), t(78)=−1.19 , p=.237 , $d_{z}=0.13$ .^3^ These results indicate that speaker repeats (vs. changes) lead to a stronger transfer within S-R sets, whereas the results do not indicate a stronger transfer between S-R sets for speaker repeats (vs. changes).

For CEs in ERs, there were significant main effects of n−1 *congruency* (CSE=2.7−0.7=2.0%) , F(1,78)=23.71 , p<.001 , $\eta_{p}^{2}=.23$ , *speaker transition* (repeat: 2.1%, change: 1.3%), F(1,78)=4.53 , p=.036 , $\eta_{p}^{2}=.05$ , and *S-R set transition* (repeat: 1.1%, change: 2.2%), F(1,78)=9.91 , p=.002 , $\eta_{p}^{2}=.11$ . Moreover, only the two-way interaction between n−1 *congruency* and *S-R set transition* was significant (S-R set repeat: 3.8% CSE, change: 0.2% CSE), F(1,78)=18.69 , p<.001 , $\eta_{p}^{2}=.19$ (both other *p*s > .538).

Finally, the three-way interaction was significant, F(1,78)=5.45 , p=.022 , $\eta_{p}^{2}=.07$ , which again reflects a larger CSE modulation by speaker transition for S-R set repeats (vs. changes). For S-R set repeats, the CSE was significantly larger for speaker repeats, (4.9%; t(78)=6.83 , p<.001 , $d_{z}=0.77$ ), than for speaker changes, (2.7%; t(78)=3.89 , p<.001 , $d_{z}=0.44$ ), t(78)=2.53 , p=.014 , $d_{z}=0.28$ . For S-R set changes, the CSE did not differ significantly between speaker repeats, (–0.4%; t(78)=0.42 , p=.679 , $d_{z}=-0.05$ ), and speaker changes, (0.7%; t(78)=0.71 , p=.481 , $d_{z}=0.08$ ), t(78)=−0.76 , p=.449 , $d_{z}=0.09$ .

For the analysis of the ${\mathrm{CSE}}_{n-2}$ , we additionally removed the second trial from each block and trials with a choice error in trial n−2 (5.6%). The resulting CEs in mean RTs and ERs were subjected to separate two-way repeated-measures ANOVAs with the factors n−2 *congruency* (congruent vs. incongruent) and *speaker transition* (repeat vs. change). For CEs in mean RTs, there was a significant main effect of n−2 *congruency* ${\mathrm{(CSE}}_{n-2}=110-81=29\;\mathrm{ms})$ , F(1,78)=41.24 , p<.001 , $\eta_{p}^{2}=.35$ . This indicates a transfer of control adjustments across S-R sets from trial n−2 to trial n . No other effect was significant (both *p*s > .107). In particular, the interaction did not reach significance (speaker repeat: 36 ms ${\mathrm{CSE}}_{n-2}$ , change: 21 ms ${\mathrm{CSE}}_{n-2}$ ), F(1,78)=2.65 , p=.108 , $\eta_{p}^{2}=.03$ . This does not indicate that the transfer of control adjustments was stronger for speaker repeats (vs. changes). For CEs in ERs, there was no significant effect (all *p*s > .365).

In summary, Exp. 2 replicated the key pattern of Exp. 1, showing again transfer of cognitive control adjustments (as reflected in a confound-minimised CSE) that was not reliably modulated by whether the speaker gender repeated or changed. However, as in Exp. 1, this speaker transition did modulate the CSE when memory-based learning processes likely contributed to the transfer between processing episodes (i.e., in confounded trial transitions).

## General discussion

In the present study, we conducted two preregistered experiments to examine the transfer of control adjustments between distinct S-R sets (as measured by the confound-minimised CSE in a prime-probe task) within and between abstract contexts defined by the speaker’s gender. Across both experiments, the confound-minimised CSE was consistently found to be not reliably affected by whether speaker gender repeated or changed and to persist for speaker gender changes.^4^ In contrast, we always observed that the confounded CSE was larger when the speaker gender repeated as compared to changed. This pattern of results also held true when considering the trial transition n−2→n (i.e., the ${\mathrm{CSE}}_{n-2}$ ). The present results thus allow a clear-cut interpretation: in the absence of S-R repetitions (i.e., between S-R sets), the transfer of cognitive control adjustments does not differ meaningfully within and between abstract contexts defined by speaker gender. However, when allowing for S-R repetitions and thereby enabling a stronger involvement of associative learning (i.e., within S-R sets), the transition of this abstract contextual stimulus feature affects the transfer of control adjustments and/or S-R-related binding and retrieval processes.

The discrepant results for confounded and confound-minimised trial transitions could reflect at least two causes. First, speaker gender might be unable to form associations with cognitive control and instead only influence the binding and retrieval of lower-level S-R features. Second, speaker gender might be able to form associations with cognitive control, but only when other associative learning processes are advantageous (here: only in the presence of S-R repetitions). Either way, the transfer of control adjustments from one 2AFC prime-probe task to another (i.e., between S-R sets) was not reliably affected by the speaker transition. This is at odds with several recent studies showing that the transfer of control adjustments between S-R sets is affected by the transition of various context features such as stimulus modality (Grant et al., 2020; Grant & Weissman, 2023; Kelber et al., 2023), stimulus format (Dignath et al., 2019; Grant et al., 2022), stimulus position (Dignath & Kiesel, 2021), and cross-modal stimulus intensity (Kelber et al., 2023).

Thus, it seems that, unlike concrete contextual stimulus features (e.g., modality and format), more abstract contextual stimulus features (e.g., speaker gender) are barely or not at all able to influence the transfer of control adjustments—perhaps because CSE modulations by context transition reflect adaptations of processing strength in lower-level perceptual channels or because these effects require context information to be available early on in order to bias conflict processing. This interpretation raises the question why previous studies found that the confound-minimised CSE was larger for repeats (vs. changes) of cross-modal intensity (Kelber et al., 2023), and that it was present when the overall S-R mapping repeated but not when it changed (Grant & Weissman, 2023). However, it seems reasonable to assume that both the emergence of statistical cross-modal correspondences and the retrieval of S-R mappings precede reasoning about the speaker gender. Thus, on this interpretation, it is insufficient to dichotomise context features as strictly concrete or abstract. Instead, it may be more appropriate to view context features along a continuum, where the degree of abstractness increases with the level at which the context feature is processed.

Furthermore, it is also possible that the transfer of control adjustments is generally less sensitive to the transition of auditory compared to visual contextual stimulus features. That interpretation is currently difficult to evaluate, however, because this study was to our knowledge the first that examined whether the confound-minimised CSE is affected by the transition of an auditory contextual stimulus feature. Nevertheless, the currently available results already offer some evidence that auditory conflict processing differs from visual conflict processing. Specifically, CEs decrease with response speed in visual prime-probe tasks (Kelber et al., 2023), indicating that distractor-based activation already fades out during target processing in vision (e.g., Burle et al., 2005; Mackenzie et al., 2022; Ulrich et al., 2015), whereas CEs increase with response speed in auditory prime-probe tasks (see Footnote 2), indicating that distractor-based activation still rises during target processing in audition. Similarly, CEs tend to increase (decrease) in auditory (visual) Simon tasks (e.g., D’Ascenzo et al., 2018). This modality difference in the time course of conflict processing seems to suggest that cognitive control operates differently in visual and auditory conflict tasks.

In general, the present findings seem difficult to reconcile with the present form of the emerging control-retrieval account (e.g., Dignath et al., 2019; Egner, 2014; Jiang et al., 2015; Schumacher & Hazeltine, 2016; Spapé & Hommel, 2008; Verguts & Notebaert, 2009). Following this account, cognitive control is bound to the context in trial n−1 and retrieved upon repetition of the context in trial n even if there are no S-R repetitions. Therefore, this account predicts a larger confound-minimised CSE for context feature repeats (vs. changes). However, there was no evidence in line with this prediction for any of the confound-minimised trial transitions in the present study. Moreover, the response that the speaker gender was simply not salient enough to affect binding and retrieval processes and thus performance is blocked by the fact that the speaker transition modulated the CSE for all confounded trial transitions. Hence, the present findings call for a refinement of the control-retrieval account to determine more precisely when and why the transfer of control adjustments is guided by the transition of (1) increasingly abstract and (2) auditory contextual stimulus features (e.g., spoken language).

The present findings also have implications for more specific accounts of CSE modulations by context transitions, namely the task-set account (Grant et al., 2020; Hazeltine et al., 2011; Schumacher & Hazeltine, 016) and the attentional-reset account (Kreutzfeldt et al., 2016). According to the task-set account, the transition of a context feature (here: speaker gender) modulates the CSE when different levels of this context feature (here: female and male) are associated with different task sets. In line with this, previous confound-minimised prime-probe task studies found a CSE modulation by context transition when the distractor context feature was predictive of the target context feature (Dignath et al., 2019; Dignath & Kiesel, 2021; Grant et al., 2020, 2022; Grant & Weissman, 2023; Kelber et al., 2023), but not when the distractor context feature was not predictive of the target context feature (Grant et al., 2020, 2022) or when the context feature belonged to a task-unrelated stimulus (Kelber et al., 2023). However, this account seems to require some elaboration in order to explain the failure to detect a modulation of the confound-minimised CSE by speaker transition in this study, given that the distractor context feature was perfectly predictive of the target context feature. Following up on the points raised above, it is possible that the formation of context-based task sets depends on the degree of abstractness and on the modality of the contextual stimulus feature.

According to the attentional-reset account, the CSE is eliminated when salient perceptual context features change. However, in the present study, the CSE persisted from trial n−2 to trial n even when the context changed twice (as well as despite two S-R set changes in Exp. 1 and despite one S-R set change in Exp. 2). Thus, this account, too, seems rather ill-suited to explain the present data. Instead, the present results seem better described by a multiple-traces account, according to which (1) separate memory traces from previous trials are maintained concurrently, (2) but only for the most strongly reactivated memory trace, context similarity guides the retrieval of the other features in this memory trace. Although the most activated memory trace T likely stems from the most recent episode (i.e., from trial n−1 ) due to fading activation, this need not be the case. For instance, in Exp. 1, $T_{n-2}$ might have overshadowed $T_{n-1}$ , partially because of the predictable S-R set and response hand repetitions (alternations) between trial n−2 (n−1) and trial n . However, we recently observed significant CSE modulations by context transition from trial n−1 to trial n with the same S-R set transitions but other contextual stimulus features (Kelber et al., 2023). As already discussed, these discrepant results might be due to speaker gender being an auditory stimulus feature and/or too abstract to allow binding and retrieval of cognitive control. In any case, the present ${\mathrm{CSE}}_{n-2}$ findings demonstrate that aftereffects of episodic processing can extend beyond the processing time of another trial. Such influences of trial n−2 on performance in trial n certainly deserve more attention in further research, such as in the current study by Schiltenwolf et al. (2023).

In conclusion, across two preregistered experiments, the CSE was reliably modulated by the transition of a more abstract contextual stimulus feature, speaker gender, when S-R repetitions were allowed but not when they were precluded. This discrepancy held true for the trial transitions n−1→n and n−2→n alike. These results suggest that the transfer of control adjustments between S-R sets takes place irrespective of the speaker transition. Instead, it seems that the speaker transition affects (1) the transfer of control adjustments only when other associative-learning processes are sufficiently at play within a single S-R set or (2) only binding and retrieval of S-R features. To accommodate the present findings, the emerging control-retrieval account seems to require some elaboration with regard to the role of abstract contexts and/or the working of auditory conflict processing, as well as with regard to the maintenance of multiple memory traces.

On a broader level, this study also provides further insights about the role of abstract representations for the transfer across different processing episodes. First, adjustments of abstract cognitive control parameters involved in conflict resolution can transfer from learning episodes to test episodes despite controlling for associative-learning processes by switching between distinct stimuli and responses and also despite intervening episodes. Second, when associative-learning processes are sufficiently at play, transfer can be guided by whether abstract information inferred about the task-irrelevant context match or mismatch between learning and test episode. Based on this, it seems worthwhile to examine in future studies whether the present conclusions about transfer and the contribution of abstract context representations generalise to other more abstract context features (such as language), and to other paradigms (such as task switching) that may rely on different control processes.

## Notes

1. For Exp. 1, we preregistered to conduct three-way ANOVAs based on mean RTs and ERs (with the additional factor n *congruency*). However, because we wanted to avoid reporting a complex four-way ANOVA in Exp. 2 (due to the addition of another factor), we decided to report ANOVAs on CEs in mean RTs and ERs consistently for both experiments (e.g., Bausenhart et al., 2021; Bräutigam et al., 2024). It should be noted that we reached the same conclusions when conducting three-way ANOVAs in Exp. 1 (and likewise, when conducting four-way ANOVAs in Exp. 2).

2. Besides analysing the CSE_*n*–1_ and the CSE_*n*–2_ at the mean level, we also scrutinised these effects at a more fine-grained distributional level. Specifically, we fitted a line to the RT delta plot, which shows the CE as a function of RT, for each participant and condition. The results of the distributional analyses of both experiments were consistent with recent findings (Kelber et al., 2023). First, CEs increased with RT in the auditory prime-probe task. Note that CEs typically decrease with increasing RT in visual prime-probe tasks. Conflict processing thus seems to differ between vision and audition. Second, significant CSEs and CSE modulations by speaker transition at the mean level were always reflected in significant intercept differences (all p s < .019), but never in a significant slope difference (all p s > .064). In other words, the pattern observed at the mean level is robust to time-varying CE fluctuations across the RT distribution (cf. Mittelstädt et al., 2022; Mittelstädt & Miller, 2020). The CSE and the CSE modulation by speaker transition thus seem to be related to adjustments of the strength rather than the timing of cognitive control (see also Mittelstädt et al., 2023). More generally, the delta plot analyses of this and the following experiment suggest that the general pattern of results (e.g., significant confound-minimised CSE that is not further modulated by speaker transition) held true even at a more fine-grained distributional level.

3. One might suspect that speaker transition affected the CSE for S-R set repeats but not for S-R set changes simply because the CSE was larger for S-R set repeats (vs. changes), thus providing more opportunities for any modulation to materialise. In this view, the CSE size should be positively correlated with the size of the CSE modulation by speaker transition. We thus checked whether the participants’ mean CSE collapsed across speaker repeats and changes was correlated with their CSE modulation by speaker transition. This was neither the case for S-R set repeats (.07), t(77)=0.64 , p=.521 , nor for S-R set changes (.07), t(77)=0.64 , p=.525 . Accordingly, the generally larger CSE for S-R repeats (vs. changes) does not seem to be a critical determinant of the discrepant results.

4. Note that in both experiments, the confound-minimised CSE was even numerically larger for speaker changes than for speaker repeats. Moreover, when analysing the combined data from both experiments (128 participants) with respect to the confound-minimised CSE_*n*–1_ to increase statistical power, the pattern remained similar. Specifically, as in both individual experiments, the combined analysis revealed a significant confound-minimised CSE_*n*–1_ that was not significantly modulated by the speaker transition.
